# Optimization of Hybrid Fiber-Reinforced Concrete for Controlling Defects in Canal Lining

**DOI:** 10.3390/ma17164000

**Published:** 2024-08-12

**Authors:** Ali Rehman, Majid Ali

**Affiliations:** Civil Engineering Department, Capital University of Science and Technology, Islamabad 45750, Pakistan; engr.dr.alirehman@outlook.com

**Keywords:** hybridization with artificial and natural fibers, polypropylene fiber, jute fiber, canal lining, mechanical properties

## Abstract

Losses in irrigation canals occur during the process of water transportation. In irrigation conveyance water losses, seepage loss is the main contributor to total water loss. The most problematic factors are cracks and settlement of the lined canal in canal lining structures. Water loss occurs in earth channels, mainly due to erosion and the permeability of the material. The concrete, as it does not present cracks, will have a less impermeable layer. Usually, seepage loss comprises 20–30% of the total water loss, and it can be reduced to 15–20% with canal linings. By enhancing the flexure and split tensile strength of concrete, the rate of cracking in the canal lining can be controlled. Concrete’s split tensile strength is one of the most important factors in crack control. The behavior (compressive, flexural, and split tensile properties, water absorption, linear shrinkage mass loss, etc.) of hybrid polypropylene and jute fiber-reinforced concrete (HPJF-RC) for the application of canal linings was studied. In this experimental work, a total of nine mixes were made with different lengths and contents of hybrid polypropylene and jute fiber-reinforced concrete (HPJF-RC) and a control mix. The SEM analysis was performed to explore the hybrid fiber cracking mechanism and the bonding of fibers with the concrete. The crack arresting mechanism of the HPJF-RC will help to reduce water losses in concrete canal linings. With this modern material, the water losses in canal linings can be minimized. The results of this experimental work would be helpful as a reference for both industry experts and academic researchers interested in the advancement of HPJF-RC composites.

## 1. Introduction

Although concrete is a commonly used building material, its poor ductility, high brittleness, low tensile strength, low tensile strain, and weak impact toughness provide numerous constraints in engineering applications [1,2,3,4,5,6]. Seepage is the term for the downward movement of water from an irrigation channel or reservoir into the soil or substratum [7]. Compared with the other types of water losses from canals, seepage (20–30%) represents a significant loss of water [8,9]. The category and amount of losses in canals varies from 25% to 60% due to conveyance losses in unlined canals [10]. In western Greece, seepage caused an average loss of flow of 17.5% per km of irrigation canals. Therefore, it is crucial to minimize this seepage loss in order to raise the conveying efficiency [11]. Water loss occurs in earth gutters, mainly due to erosion and the permeability of the material. As it does not present cracks, concrete will have a less impermeable layer. A reduction in seepage losses by almost 39% could be achieved by providing lining to irrigation minors [12]. Because concrete materials are typically accessible in the surrounding areas of the local farmers, concrete is frequently used for canal linings to minimize seepage loss [13]. The structure of the concrete canal lining is the same as that of a thin plate with frequent cracking [14,15]. Significant seepage (15–20%) is present, even in the conventional cement–concrete sections. Water loss can be decreased with the use of concrete canal linings for enhanced performance [15,16]. Differential foundation settlement, external loads, and thermal stress are just some possible causes of these cracks [15,17].

Commercially, a wide variety of fibers are available, such as steel, glass, synthetic, and certain natural fibers. Polypropylene fiber is one of the best micro-reinforced materials available nowadays for enhancing concrete performance [18,19,20,21,22]. Fibers have been used extensively in concrete all over the world, according to the ACI Committee report on the worldwide application of fibrous concrete. Fiber-reinforced concrete has, in fact, been used to create a number of significant and fascinating projects, including runway slabs and parking garages with demountable panels [23]. Because of its minimal environmental effect and superior properties when hardened over conventional concrete, massive amounts of various types of fiber-reinforced cement are produced every year [24]. Indeed, fiber-reinforced concrete (FRC) offers a variety of technical benefits, such as the ability to control cracks, higher flexural strength, adequate failure impact resistance, and lower permeability, in addition to reducing the expansion and shrinkage rate of concrete [25,26,27]. In fact, the effect of fibers on fresh concrete properties is determined by their nature, shape, length, and volume fraction [28].

Jute fiber’s impact as a reinforcing component in concrete was investigated. Standard-sized specimens were tested for their tensile, compressive, and flexural strengths. The incorporation of jute fibers resulted in a significant improvement in mechanical properties. Jute fiber-reinforced concrete was shown to be a potentially useful material for the construction of low-cost concrete [29]. Research was conducted on the efficiency of jute fibers in cement paste and mortar. It was determined that jute fiber-reinforced concrete (JFRC), particularly in the South Asian region, could be a cost-effective and economical material for building construction [30]. Investigations were conducted on concrete reinforced with coir, bamboo, and jute fibers for both mechanical and impact loadings. A 15 mm long fiber with a 1% volume fraction was utilized. When jute and bamboo fiber-reinforced concrete were compared with PC, a decrease in compressive strength was noted [31]. Researchers and engineers will be able to optimize the mixture design and estimate the C-S of FRC for various engineering applications with the help of this precise and adaptable property assessment tool provided by the suggested deep-learning approach [32]. Steel macro-fiber and polypropylene micro-fiber hybrids showed some synergistic effects. On the other hand, maximum synergy was demonstrated when carbon and polypropylene microfibers were hybridized with crimped polypropylene macro-fibers [33].

These inherent shortcomings of concrete can be enormously enhanced by employing the idea of micro-level reinforcement, i.e., adding different discontinuous chopped fibers into concrete [34]. It is important to note that polypropylene fibers offer several benefits over other synthetic fibers that are available commercially, including being less expensive, having low heat conductivity, being lightweight, and being resistant to alkali and acid attacks. Moreover, the hydrophobic levels in polypropylene fibers shield them from cement paste wetting and do not change the amount of water required. As a result, the use of PF in field concrete is becoming more popular these days. In addition, it has been reported that adding polypropylene fibers has a low impact on air content and fresh density because of the low density of PF, unlike other types of fibers such as steel fibers [35]. The initial occurrence of cracks in concrete structures frequently leads to their gradual deterioration or destruction, ultimately resulting in a contraction of their effective loading area [36]. Concrete structure cracking is primarily caused by the properties of the concrete material. The deterioration and corrosion of steel reinforcement in a harsh atmosphere are caused by the cracking in concrete. Corrosive chloride ions and carbon dioxide enter the concrete through these cracks and reach the reinforcement. The concrete that is not cracked prevents water from penetrating, in contrast to the concrete that is cracked [37]. Composites made of ordinary cement are recognized for being brittle and prone to sudden failure. Their brittleness results in comparatively low tensile strength and reduced resistance to cracking [38]. Adding hybrid fibers to cement-based composites increases their tensile strength and resistance to cracking [39]. In order to strengthen and toughen cement-based composites, fibers containing multi-scale properties are also widely utilized. In cementitious composites, the use of both short and long fibers can restrain the cracking at different levels [38]. Small cracks that appear to be very minor at first may eventually grow and lead to structural failure. Concrete first develops a micro-crack, which subsequently undergoes meso-crack and macro-crack transformations. Whether a crack originates from a micro-, meso-, or macro-crack, it affects the structural integrity and mechanical behavior. The concrete structures’ actual strength will ultimately be diminished by the occurrence of these types of cracks [40].

Water loss occurs in earth channels, mainly due to erosion and the permeability of the material. As concrete does not present cracks, it will have a less impermeable layer. The cracking rate in concrete canal linings can be controlled by improving the concrete’s flexural strength and split tensile strength. One of the key elements in crack control is the split tensile strength of concrete [41,42]. The detailed behavior of hybrid polypropylene and jute fiber-reinforced concrete (HPJF-RC) for its application in canal lining, including its compressive, flexural, and split tensile properties, water absorption, linear shrinkage, mass loss, etc., has not yet been studied. In this experimental work, a total of nine mixes were made with different lengths and contents of HPJF-RC and a control mix. An SEM analysis was performed to explore the hybrid fiber cracking mechanism and bonding of the fibers with the concrete. Further properties determined for HPJF-RC included its strength and energy absorption, and the toughness index under compressive, flexural, and split tensile load was calculated. Moreover stress–strain curves, compressive strength, and load-deflection curves for the flexural and split tensile strength were determined. Usually, the natural or mineral fibers help to restrict the macro- or meso-crack. Therefore, a useful, cost-effective, and environmentally friendly material to be used in some important structures to resist impact loads is jute fiber-reinforced concrete with steel rebars [43]. The main objective of the research program is to minimize water loss in canal linings by using advanced materials, such as a hybrid fibers (polypropylene and jute fibers) compared with adding a single type of fiber, and also to improve mechanical properties, water absorption, and linear shrinkage.

## 2. Experimental Scheme

### 2.1. Raw Ingredients of Concrete

For the production of the normal control mix (CM), ordinary Portland cement (Bestway cement), Margalla crush, and Lawrencepur sand were used. The production of OPC from Bestway cements was as per EN 1971: 2011-CEM I 425N [44], i.e., having a 28-day strength of 52 ± 3 MPa. For the CM and the hybrid polypropylene and jute fiber-reinforced concrete (HPJF-RC), silica fume and superplasticizer were used. The silica fume and superplasticizer were bought from Sika Company in Pakistan. The maximum size of the aggregate used for manufacturing both the CM and the HPJF-RC was 19 mm. Tap water (at normal temperature) was used for preparing the CM and all the HPJF-RC. Fibers of 6 mm and 12 mm lengths and polypropylene fiber with a diameter of 20 µm were selected based upon their commercial availability. Jute fibers were taken in raw form. Firstly, the jute fibers were combed properly to achieve straight fibers for ease of cutting and further use in concrete. Then, these fibers were cut into the desired lengths of 6 mm and 12 mm with a diameter of 20–200 µm.

Polypropylene fiber is a commercially available synthetic fiber that has low heat conductivity, is lightweight, and is resistant to alkali and acid attacks. One of the cheapest locally available material and one of most durable of natural plant-based fibers is jute fiber. The properties of PF and JF are given in Table 1. It can be noted that PF has a lower density, zero water absorption, and good in tensile strength. And for the JF, its tensile strength is good enough among the other natural fibers. Researchers have used PF and JF in their experimental studies [35,43]. It can be more beneficial to utilize PF in irrigation concrete canal lining.

**Figure 1:**
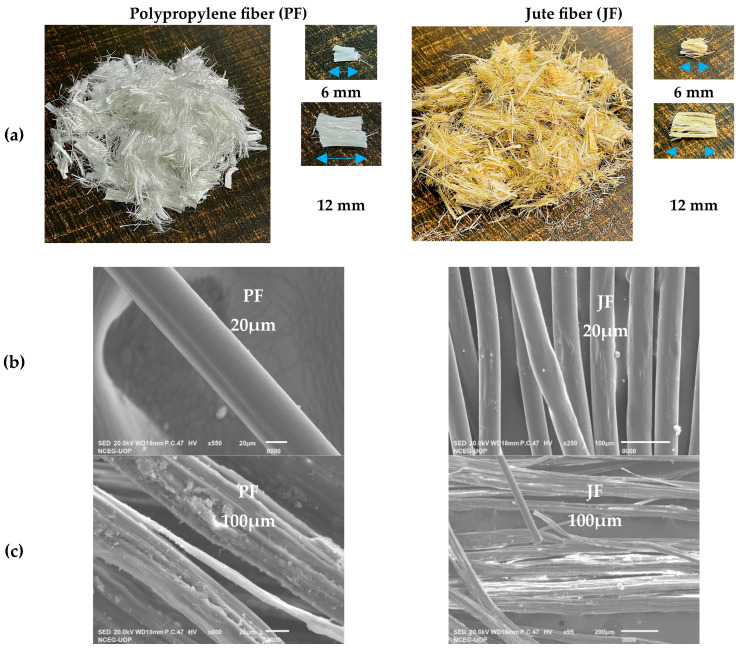
Considered fibers: (**a**) Physical representation; (**b**) SEM image @ 20 µm and (**c**) SEM image @ 100 µm.

The physical appearance of the polypropylene fiber and jute fiber provided by the manufacturers are shown in Figure 1a. The distinct variations in the shape of the polypropylene fibers are evident in Figure 1b, which was revealed through scanning electron microscope (SEM) analysis. It can be reported that jute fiber consists of microtubes, which are spread concurrently in the longitudinal orientation, as shown in Figure 1c. This empty state of the microtubes can be observed by examining the fiber’s edge. This could be the cause of the jute fiber’s ability to absorb water. Jute fibers exhibit a mean diameter of 0.1 mm, a 62% absorption rate following a 24 h immersion in water, and an average tensile strength of 61 MPa [46].

One of the most widely used pozzolanas is silica fume. When added to concrete mixtures, it reduces porosity, permeability, and bleeding because the oxides in the fume (S_i_O_2_) react and consume calcium hydroxides, which are created when regular Portland cement hydrates. Reduced heat release and strength development, lime-consuming activity, and a smaller distribution of pore sizes are the primary outcomes of pozzolanic reactions [47]. The chemical composition of cement and silica fume are shown in Table 2.

### 2.2. Mix Design and Casting of Specimens

For the production of the CM mix, all of the raw ingredients were combined in a mixer for two minutes. A layer process for mixing fiber-reinforced concrete was used to produce the HPJF mix in its entirety. The literature also reports using this technique to mix H-FRC properly and to prevent the balling formation of fibers [48,49]. After uniform mixing, six cylinders of 100 mm diameter and 200 mm height and three beam specimens of 100 mm width, 100 mm depth, and 457 mm length were cast from each batch. The fresh concrete mix was poured into the plastic molds, and then compaction was performed by steel rods. After 24 h, the cylinders and beams were demolded and kept for 28 days in the curing tank. The ASTM-C192 was followed for making and preparation of specimens [50].

The representation of mixes and their 1:2:3 proportion with a w/c ratio of 0.40 for all the concretes mixes are shown in Table 3, and this proportion has also been reported in the literature [51,52,53,54,55]. Silica fume was also utilized at 10% of the cement mass in the HPJF-RC; this percentage was also reported by [56,57,58,59] and showed a positive impact on the hybrid fiber concrete composites.

A total of nine specimens were cast from each batch of the CM, HPJF-1 (0.5 + 0.5% and 6 + 6 mm), HPJF-2 (0.25 + 0.75% and 6 + 6 mm), HPJF-3 (0.75 + 0.25% and 6 + 6 mm), HPJF-4 (0.5 + 0.5% and 12 + 12 mm), HPJF-5 (0.25 + 0.75% and 12 + 12 mm), HPJF-6 (0.75 + 0.25% and 12 + 12 mm), HPJF-7 (0.5 + 0.5% and 6 + 12 mm), HPJF-8 (0.25 + 0.75% and 6 + 12 mm), and HPJF-9 (0.75 + 0.25% and 6 + 12 mm). HPJF-1 to HPJF-9 represent hybrid fiber-reinforced concrete containing polypropylene and jute fibers different contents and fiber lengths at a total of 1% of the concrete mass. These hybrid fiber contents and lengths have also been studied [60,61,62,63]. The slump test was performed as per ASTM-C143 [64] and ACI-Committee-544.1R-96 [46]. Table 3 also demonstrates the slump values and hard densities of the CM and all HPJF-RC mixes. The inclusion of the hybridization of polypropylene and jute fibers resulted in low slump values. For the CM, the slump value was the maximum compared with HPJF-RC. The slump values were lower for the mixes having a higher percentage of jute fiber at its maximum length. The density was calculated according to ASTM C138/C138M-16 [65]. It was observed that the maximum value density value was achieved by the CM. And all other combinations of the hybrid fiber-reinforced concrete achieved a lower density compared with the CM.

### 2.3. Dynamic Testing

Before the destructive (mechanical) testing of the specimens, dynamic testing was performed as per ASTM C215-14 [66]. In dynamic testing, the longitudinal resonant frequencies (LRFs), transverse resonant frequencies (TRFs), and rotational resonant frequencies (RRFs) are determined with the use of a hammer and an accelerometer. The test is performed on cylinders and beamlets. For determining the LRF, the accelerometer is connected to one side of the cross-section of cylinders and beamlets while a strike of the hammer is given to the opposite side of the cross-section of the specimen.

The accelerometer observes the frequencies and transfers the record of these frequencies to the computer connected with it. The procedure for attaching the TRF and RRF to the accelerometer and the strike location of the hammer are different for the cylinders and the beamlets. In the case of the cylinders, for the TRF, the accelerometer is connected at the side facing the length of the cylinder from at least 250 mm away but before the edge. Then, a strike of the hammer is given at the same side facing the center of the cylinder’s length.

For the RRF, the accelerometer is attached at the top facing the length of the cylinder with same spacing from the edge as for the TRF. The strike is given perpendicular to the accelerometer on the opposite edge of the cylinder length. In the case of the beamlets, for the TRF determination, the accelerometer is connected at one side of the length of the beamlets at the same edge used for the cylinders. A small hammer strike is given at the center of the length on the same side to which accelerometer is connected.

For the RRF, the accelerometer is attached at the top corner of the rectangle side face of the beamlet. A strike is given at the other side at the bottom corner of the same side of the rectangle in such a way that the line joining the point of the hammer’s strike and the accelerometer makes the diagonal of the rectangle. From these observed resonant frequencies, the dynamic modulus of elasticity and the damping ratio are calculated. These calculated properties aid in understanding the behavior and resistance of the CM and all batches of the HPJF-RC against dynamic loading.

### 2.4. Mechanical Testing

#### 2.4.1. Compression Test

A servo-hydraulic testing machine (STM) was used for the determination of the compressive strengths of PC and all the HPJF-RC. The test was performed according to ASTM C39 [67] on cylinders of the CM and HPJF-RC. In this test, the properties determined included the compressive strength (C-S), stress–strain curves, pre-crack energy absorption of compressive property (CPEA_1_), post-crack energy absorption of compressive property (CPEA_2_), total energy absorption of compressive property (CTEA), and toughness index of compressive property (C-TI) of the CM and HPJF-RC. To distribute the load uniformly throughout the cylinder, the capping of the cylinder was prepared with plaster of Paris material.

#### 2.4.2. Flexural Test

The flexural test was performed on the basis of ASTM C78 [68]. The three-point loading mechanism was adopted. The test was performed on beamlets of the CM and HPJF-RC. The studied parameters in this test were the curves of load deflection, flexural strength (F-S), flexural pre-crack energy absorption of flexural property (FPEA_1_), post-crack energy absorption of flexural property (FPEA_2_), total energy absorption of flexural property (FTEA), and toughness indexes of flexural property (F-TIs).

#### 2.4.3. Split Tensile Test

ASTM C496 standard was used for the split tensile test [69]. The same STM machine was used for performing the test. The test was performed on cylinders of the CM and HPJF-RC. The studied parameters in this test were the curves of load deflection, split tensile strength (S-T-S), pre-crack energy absorption of split tensile property (SPEA_1_), post-crack energy absorption of split tensile property (SPEA_2_), total energy absorption split tensile property (STEA), and toughness indexes of split tensile property (S-TIs).

### 2.5. Miscellaneous Testing (Water Absorption, Linear Shrinkage, and Mass Loss)

For the calculation of the water absorption properties of the CM and HPJF-RC, the ASTM C642 [70] standard was considered. First of all, specimens were dried of water in the oven, and then these dried specimens were placed, at room temperature, in water. This method was applied to determine the water absorption of the CM and HPJF-RC specimens. For the calculation of linear shrinkage, ASTM C157/C157M-08 [71] was followed by observing and measuring the variations in the length of the CM and HPJF-RC specimens.

For this purpose, a line of 6 inches was marked as a reference on the length of specimens before conducting the test. The variation in the length is measured after following the procedure for the standard. The linear shrinkage is then measured by taking the percentage difference of the marked length before and after the test procedure.

ASTM C157M-08 was used for the determination of mass loss in the CM and HPJF-RC. After following the test procedure, shrinkage and variation in the reference line were marked before being calculated. Specimens from each batch of CM and HPJF-RC were placed in a high-temperature heating oven. The temperature was raised from 20 °C to 100 °C at a rate of increase of 3 °C per minute and maintained at 100 °C for one hour. This was performed to obtain more realistic data. Then, the specimens were cooled down at the same rate of decrease in temperature of 3 °C per minute to avoid thermal cracking of all the CM and HPJF-RC specimens.

### 2.6. SEM Analysis Procedure

Fiber bridging, pull-out, and rupture are the three primary crack-resisting mechanisms of HPJF-RC. There are different types of fiber that can experience pull-outs, bridgings, and ruptures. According to reports, the fracture performance of fiber-reinforced composites (FRCs) is directly impacted by fiber rupture and pull-out, while the performance of crack bridging is dependent on it. Therefore, improved fiber–matrix bonding is crucial for increasing ductility [72]. The study also demonstrated how fibers, through their interfacial debonding with the matrix, are essential for bridging cracks and preventing their propagation [73]. Moreover, fiber pull-out in the case of normal- or low-strength concrete is the most crucial step in increasing the fracture energy in FRCs [74].

## 3. Results and Analysis

### 3.1. Dynamic Properties of Concrete Composites

Concrete composite dynamic properties of the CM and HPJF-RC were investigated to evaluate the influence of the hybridization of polypropylene and jute fibers lengths on the properties of concrete specimens. Dynamic properties of CM samples were obtained by the ASTM standard C215-14. Due to unavailability of a specific standard to determine the dynamic properties of the HPJF-RC, the same standards were adopted to determine the dynamic properties of the HPJF-RC.

Additionally, fibers were utilized to enhance the dynamic properties of concrete. An investigation was conducted into the dynamic properties of cylinders made of coir fiber-reinforced concrete (C-FRC). Resonance frequencies and the dynamic elastic modulus of unconfined C-FRC were found to be lower than those of plain concrete [75]. The researcher also studied the dynamic properties of coconut fiber-reinforced concrete. A decrease in frequencies and an increase in damping were also noted when comparing coconut fiber-reinforced concrete with PC. When the fiber content increased, damping increased as well, and increments became more noticeable as the cracking increased [76]. Dynamic action vibrations commonly result in service issues, but in rare circumstances, they can also seriously threaten safety by risking the stability of the entire structure [77]. This fact has motivated, during the last decades, new research lines focused on the study of the dynamic behavior of new materials and composite concretes [78].

Table 4 shows the calculated dynamic properties of the CM and HPJF-RC. An average of three obtained values were taken to achieve the average and suitable results for each CM and HPJF-RC specimen combination for each corresponding dynamic property. Table 4 shows the longitudinal resonant frequencies (LRFs), transverse resonant frequencies (TRFs), rotational resonant frequencies (RRFs), and dynamic elastic modulus (Ed) of the different CM and HPJF-RC specimens. When the specimens are compared with each other, the CM has a higher Ed than the HPJF-RC cylinders and beamlet specimens. HPJF-5 shows the maximum Ed value decrease as compared with the other mixes. The dynamic property maximum value is observed for the mix of HPJF-9 cylinders and beamlet specimens.

### 3.2. Mechanical Properties

#### 3.2.1. Compressive Properties

**Figure 2:**
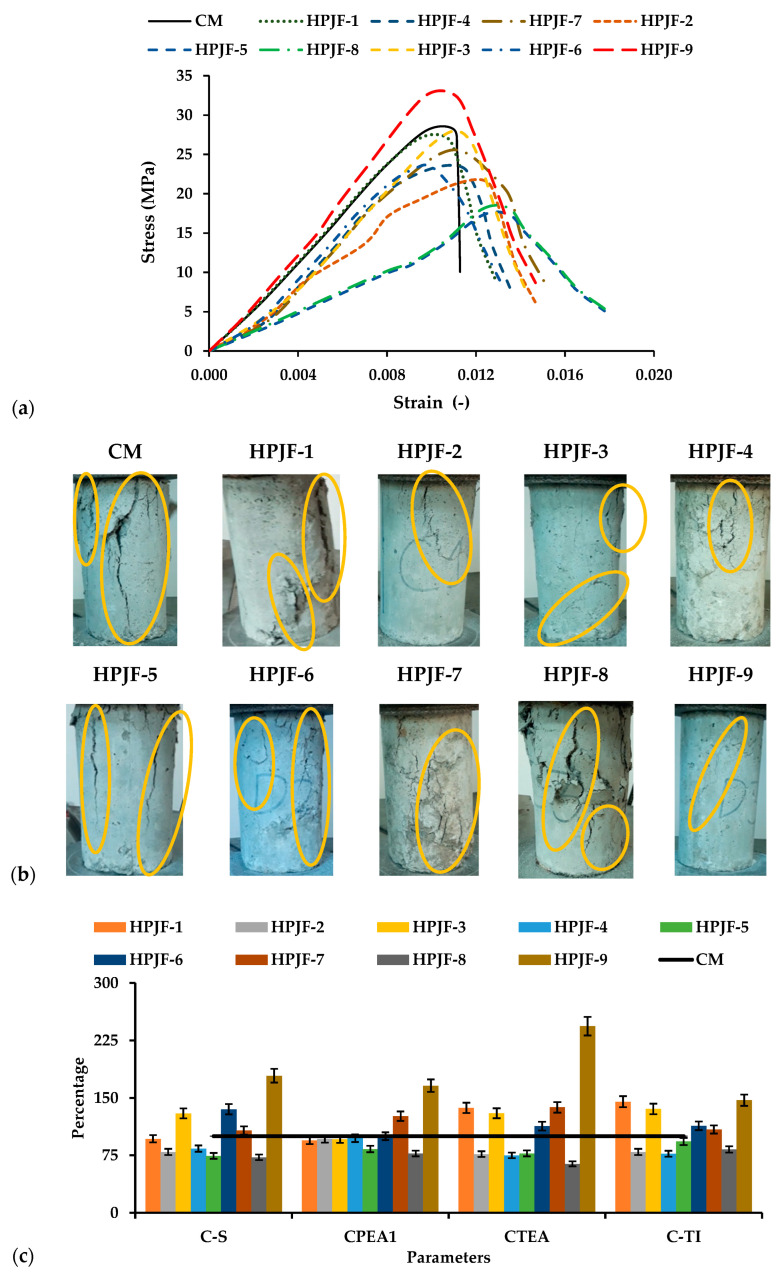
Compressive behavior of all the concrete samples: (**a**) Stress–strain curves; (**b**) Cracks in the tested specimens at peak load; (**c**) All the concrete samples in comparison with the CM.

The relationship between the stress–strain graphs of the CM, HPJF-1, HPJF-2, HPJF-3, HPJF-4, HPJF-5, HPJF-6, HPJF-7, HPJF-8, and HPJF-9 is presented in Figure 2a. In this figure, it can be noted that HPJF-9 shows the maximum value of compressive strength (C-S) in comparison with all the types of HPJF-RC and the CM. Moreover, the HPJF-9 stress–strain curve shows a higher strain capacity at the ultimate stage, with an enhanced unstiffening property after the peak stage in comparison with all the types of HPJF-RC and the CM. The behavior of the CM and HPJF-RC specimens and the growth of cracks and the cracks are marked with yellow circles are presented in Figure 2b. At peak load for the CM, the first crack was produced. In the case of the HPJF-RC, the first crack was also observed at peak load. However, cracks in HPJF-9 were very small in both length and width as compared with the cracks in the CM. The size and number of cracks increased considerably at the maximum load for the CM in comparison with the HPJF-RC. HPJF-RC only showed a slightly increase in the crack size and number. The better performance of HPJF-RC is because of the bridging provided by the hybridization of PF and JF. For the determination of the hybrid fiber’s failure mechanism, HPJF-RC specimens were purposely broken. It was noted that short length fibers arrested small cracks and that large fibers arrested large cracks. Furthermore, adding a higher percentage of fibers resulted in a more congested mix, which eventually lowers strength [79]. The comparison of all HPJF-RC specimens’ C-S in comparison with that of the CM is shown in Figure 2c. The reason for the improvement in the C-S is the silica fume effect and also the crucial crack-bridging effect of the polypropylene and jute fibers, which play a significant role in enhancing the C-S of the concrete. Also, the addition of a higher percentage of fibers results in the heterogeneity of the concrete mix, ultimately reducing its strength.

Table 5 shows the values of the C-S, pre-crack energy absorption (CPEA1) of compressive property, post-crack energy absorption (CPEA2) of compressive property, total energy absorption (CTEA) of compressive property, the toughness index (C-TI) of compressive property, and modulus of elasticity (M-E). The compressive strength of HPJF-1 to HPJF-8 were reduced by 04%, 23%, 01%, 17%, 38%, 17%, 11%, and 36%, respectively. The HPJF-9 showed the maximum value of compressive strength among all other types of HPJF-RC. When the polypropylene fiber content was increased up to 0.75% with a length of 6 mm and 0.25% JF with a 12 mm length in the HPJF-RC, the multi-scale hybrid fiber (MSHF) crack-arresting mechanism and silica fume effect on the compressive strength increased up to 15% as compared with the CM. Addition of 6 mm PF and 12 mm basalt fiber in the composite improved the C-S up to 5%, as reported by Yan et al. [80]. It has been shown that mechanical properties with hybrid basalt fiber content increase the C-S by 9.5% as compared with PC [79]. Incorporating a higher fiber percentage results in the formation of air voids and a decrease in workability, which may eventually result in less compaction and a decrease in the C-S. The lower C-S can be attributed to the reduced amount of cement that results from incorporating a higher fiber percentage into the concrete. It is also important to mention that the C-S minimum and maximum coefficient of variance percentages are 1% and 6%, respectively, as illustrated in Table 5. From the coefficient of variance (CoV), it is evident that the C-S exhibits a maximum error variability of up to 6%.

All these absorbed energies and the toughness indexes of compressive properties were calculated by defined methods [36,47,79]. The energy absorbed per unit volume of material, which is also referred to as the energy absorption ability, was described as the area under the stress–strain curve, and MJ/m^3^ are the units [81]. There was a reduction of 6%, 9%, 13%, 15%, 29%, 29%, 11%, and 31% in the values of CPEA1 for HPJF-1 to HPJF-8, respectively, when compared with the CM and a 14% increase in HPJF-9 with a 0.75% fiber content and length of 6 mm. On the other hand, the respective CPEA2 values were increased by 1.08, 3.42, 1.23, 4.20, 1.80, 4.42, 0.84, 2.09, and 1.96 times as compared with the CM.

The C-TI values for all the HPJF-RC specimens were greater than that of the CM. This increase may be due to presence of positive synergy effect of hybrid PF and JF. Diagonal and shear cracks appeared on samples of the HPJF-RC under compressive loading. HPJF-9 had the maximum C-TI values. So, HPJF-9 has a positive impact on the C-TI. The study reported that steel fiber–CaCO_3_ whisker–basalt fiber reveals a positive synergy effect delivered by MSHFs after peak stress than by other types of FRCs [79].

#### 3.2.2. Flexural Properties

**Figure 3:**
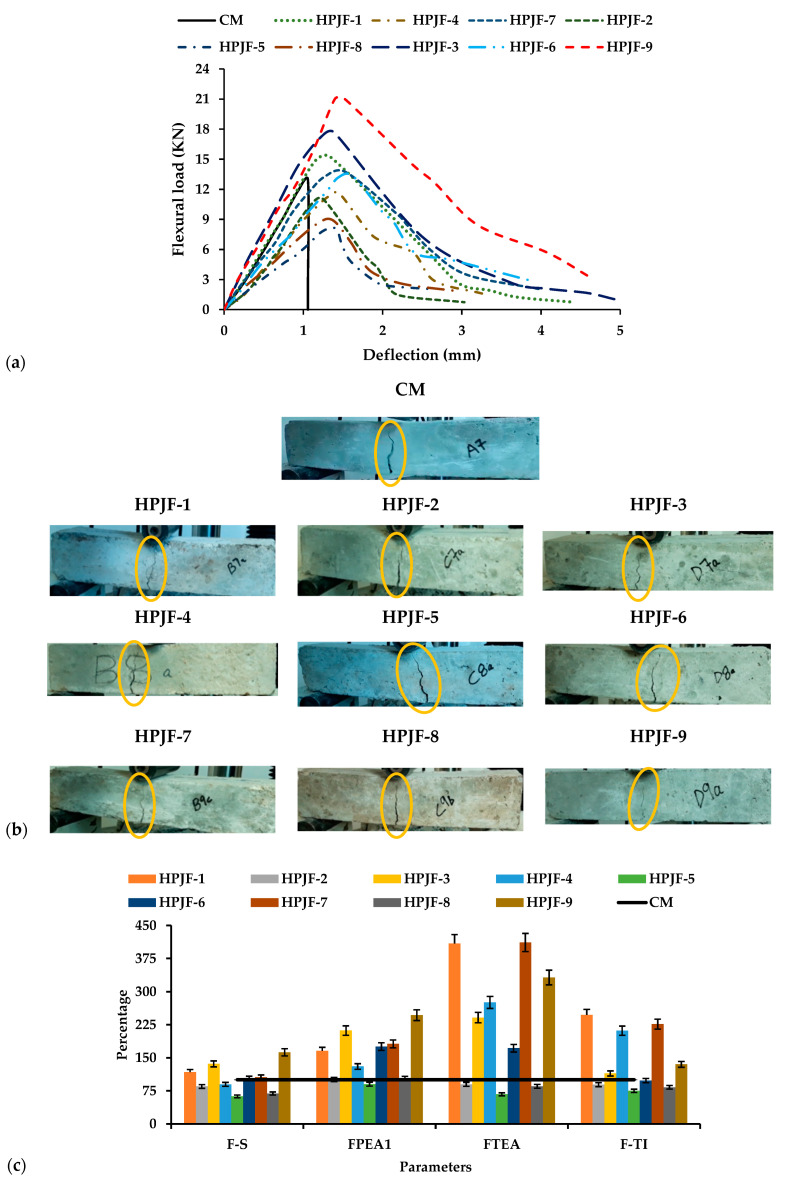
Flexural behavior of all the concrete samples: (**a**) Load-deflection curves; (**b**) Cracks in the tested specimens at peak load; (**c**) All the concrete samples in comparison with the CM.

The load-deflection curves for CM, HPJF-1, HPJF-2, HPJF-3, HPJF-4, HPJF-5, HPJF-6, HPJF-7, HPJF-8, and HPJF-9 are given in Figure 3a. According to ASTM standard C78, a loading rate of 1.03 MPa/min is recommended to be applied at testing, but in this current work, a loading rate of 1 kN/s was applied. It is noted that the HPJF-9 carries the maximum load as shown in the figure because it resists the maximum flexural loading. Due to the bridging effect of the PF and JF, the HPJF-RC shows the better load-carrying capacity. Other HPJF-RC mixes having shorter lengths with the minimum percentage of JF provide good strength as compared with those with a greater percentage and length of JF.

Observation revealed that all specimens abruptly lost strength after attaining their maximal strength. The strength of the CM specimen was zero after it broke into pieces. However, the HPJF-RC specimen was still intact and retained some strength. Figure 3b displays the behavior of the specimens and the cracks’ development and the cracks are marked with yellow circles. The first crack in the CM specimen developed immediately after it split into two pieces at maximum load. On the other hand, the HPJF-RC revealed cracks at peak load. The HPJF-RC continued to carry the load and did not break apart even at its peak load. However, the crack’s width increased to 20 mm and began propagating upward. The crack in HPJF-9 was very small in both width and length in comparison with the crack in other mixes of the HPJF-RC. After peak load, the length and width of the cracks increased in the HPJF-RC. The comparison of F-S values for all the HPJF-RC specimens in comparison with the CM is shown in Figure 3c.

The excellent performance of the HPJF-RC as compared with CM is because of the bridging effect of hybrid PF and JF-reinforced concrete mixes. PF and JF create a strong bond within the concrete matrix, and not a single piece of concrete drops down. To find out the fiber’s failure mechanism, HPJF-RC specimens were purposely broken. Fiber breakage and pull-out were noticeable from the broken surface.. It is noted that small- length fibers arrested small cracks, and the longer fibers arrested large cracks.

Table 6 shows the values of the F-S, pre-crack energy absorption of flexural property (FPEA1), post-crack energy absorption of flexural property (FPEA2), total energy absorption of flexural property (FTEA), and toughness indexes of flexural property (F-TIs). The F-S of HPJF-1, HPJF-6, HPJF-7, and HPJF-9 increased by 17%, 3%, 6%, and 62% as compared with the CM. HPJF-9 had the maximum F-S value. This may be due to the presence of the positive synergy effect of the hybridized polypropylene and jute fibers. The overall increase in F-S in all the HPJF-RC specimens is due to the fiber effect of bridging. Shorter fibers, like those of 6 mm length, arrest small cracks, and longer fibers, like those of 12 mm length, arrest larger cracks. When the PF content is increased up to 0.75% with a length of 6 mm and the JF contentof (total of 1% by concrete mass) 0.25% with a 12 mm length in the HPJF-RC, the MSHF crack-arresting mechanism and the SF effect on the F-S increased up to 62% as compared with that for the CM. The F-S decreased with an increase in JF percentage (0.75%) in the HPJF-RC. Addition of 6 mm PF and 12 mm basalt fiber in the composite improves the F-S up to 17.7%, as reported by Yan et al. [80]. The study reported that the addition of a JF length of 50 mm to the concrete improved the F-S of JFRC by 128% [43]. Combining a higher fiber percentage results in the formation of air voids and a decrease in workability, which may eventually result in less compaction and a decrease in F-S. The lower F-S can be attributed to the reduced amount of cement that results from incorporating a higher fiber percentage into the concrete. It is also important to mention that the F-S minimum and maximum coefficient of variance percentages were 4% and 8%, respectively, as demonstrated in Table 6. From the coefficient of variance (CoV), it is evident that the F-S exhibits a maximum error variability of up to 8%.

All these absorbed energies and toughness indexes of F-S were calculated by defined methods [36,47,79]. The energy absorbed per unit volume of material, which is also referred to as the energy absorption ability, was described as the area under the load-deflection curve, and Jules (J) are the units [81]. The FPEA1 of HPJF-1 to HPJF-4 and HPJF-6 to HPJF-9 were increased by 0.7, 0.01, 1.1, 0.3, 0.8, 0.8, 0.03, and 1.5 times, respectively, as compared with that of the CM. No FPEA2 occurred in the case of the CM because under peak loading, the CM broke apart into two pieces. Therefore, only HPJF-RC shows the FPEA2. The HPJF-RC may also be predicted to withstand flexural loading slightly longer than the CM. The addition of fibers to concrete serves as a crack arrestor and prevents cracks from propagating. The F-TIs of HPJF-1 to HPJF-9 were greater than that of the CM. The HPJF-9 has the maximum F-TI due to its maximum pre-crack and post-crack energies. This may be because of the incorporation of a hybrid of one longer length of JF and one shorter length of PF, whose ability to prevent cracks after flexural loading was enhanced compared with other shorter fiber combinations. The loading rate all through the test was similar. The reason behind the nonlinear pre-crack behavior of concrete is the addition of hybrids of varying fiber lengths. The improved properties are due to MSHF, i.e., polypropylene fibers, jute fibers, and silica fume, which offer crack resistance at the macro-, meso-, and micro-levels, respectively.

The following empirical relation is established between the S-T-S and C-S of the HFRC:

F-S = F × C − S(1)

where F is the conversion factor incorporating the combined effects of fiber type, length, and content.

#### 3.2.3. Split Tensile Properties

**Figure 4:**
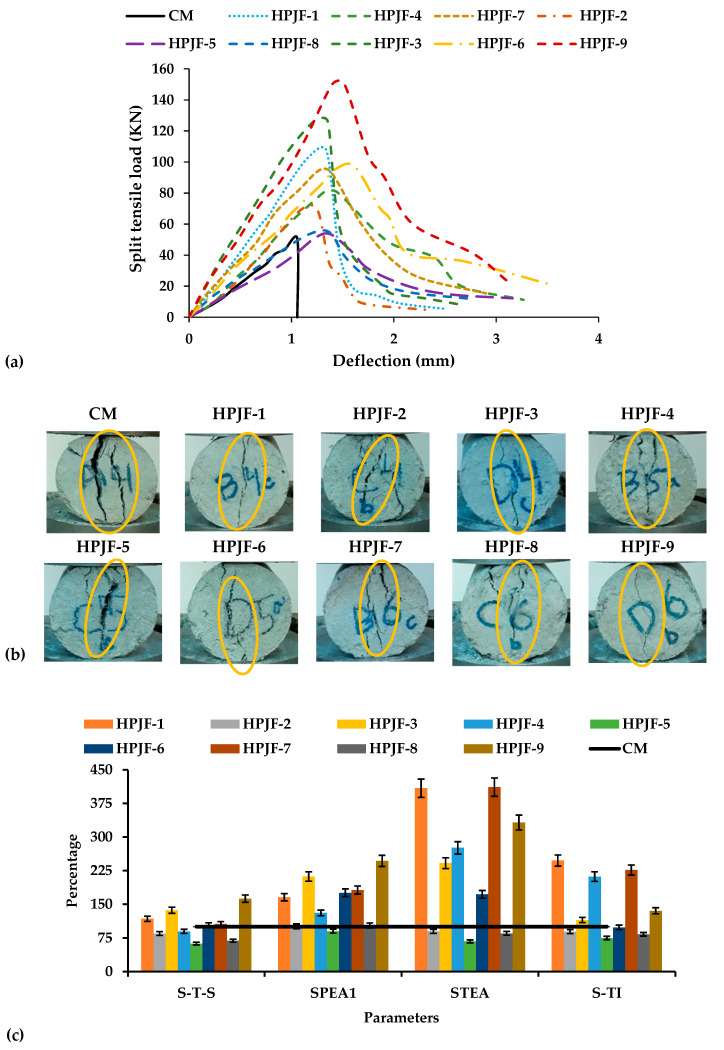
Split tensile behavior of all the concrete samples: (**a**) Load-deflection curves; (**b**) Cracks in the tested specimens at peak load; (**c**) All the concrete samples in comparison with the CM.

The graph of load deflection for CM, HPJF-1, HPJF-2, HPJF-3, HPJF-4, HPJF-5, HPJF-6, HPJF-7, HPJF-8, and HPJF-9 are presented in Figure 4a. It is noted that the HPJF-RC carries the maximum load as shown in the figure. Due to the bridging effect of the PF and JF, HPJF-RC has shown the better load-carrying capacity. Other HPJF-RC mixes having shorter lengths with the minimum percentage of JF provide good strength as compared to those with a greater percentage and length of JF. The HPJF-9 hybrid combination of polypropylene and jute fibers gives maximum strength compared with the CM and all other hybrid combinations of HPJF-RC. The HPJF-RC having the maximum percentage of PF provides better results in all aspects.

It is noted that after reaching their maximum strength, there is an abrupt decrease in the strength of the CM and all HPJF-RC specimens. The CM was broken into pieces, and its strength went down to zero. However, HPJF-RC specimens remained unbroken, and it means that still had some strength. The behavior of the specimens and growth of the cracks and the cracks are marked with yellow circles are presented in Figure 4b. The first crack in the CM specimen developed immediately after it split into two pieces at maximum load. On the other hand, cracks in the HPJF-RC were observed at peak load. HPJF-RC continued to carry the load and did not break apart even at its peak load. Cracks in HPJF-9 were very small in both length and width in comparison with the cracks in other mixes of HPJF-RC. After peak load, there was an increment in crack length and width in the HPJF-RC. HPJF-RC showed excellent performance as compared with the CM, which is because of the bridging effect of hybrid PF and JF-reinforced concrete mixes. PF and JF create a better bond within the concrete matrix, and not a single piece of concrete drops down. The comparison of the C-S of all the HPJF-RC specimens in comparison with the CM is shown in Figure 4c. For the determination of the hybrid fiber’s failure phenomena, HPJF-RC specimens were purposely broken. It is noted that in the short-length fibers arrested small cracks, and longer fibers arrested large cracks. The low tensile strength of the JF caused more damage but prevented pull-out due to its greater bond strength.

Table 7 shows the values of the split tensile strength (S-T-S), pre-crack energy absorption of split tensile property (SPEA1), post-crack energy absorption of split tensile property (SPEA2), total energy absorption split tensile property (STEA), and toughness indexes of split tensile property (S-TIs). The S-T-S of HPJF-1 to HPJF-9 was increased by 111%, 61%, 87%, 39%, 5%, 8%, 151%, 92%, and 196% as compared with that of the CM. HPJF-9 has the maximum S-T-S value. This may be due to the presence of the positive synergy effect of the hybrid polypropylene and jute fibers. The overall increase in S-T-S of all the HPJF-RC specimens is due to the bridging effect of hybrid fibers [57,76,82]. The HPJF-RC may also be predicted to withstand tensile loading for slightly longer than the CM. The addition of fibers to concrete serves as a crack arrestor and prevents cracks from propagating. Shorter fibers, like the 6 mm length, arrest small cracks, and longer fibers, like the 12 mm length, arrest larger cracks. When the PF content was increased up to 0.75% with a length of 6 mm and that of the JF to 0.25% with a 12 mm length in the HPJF-RC, the MSHF crack-arresting mechanism and the SF effect on the S-T-S increased up to 196% as compared with that of the CM. The S-T-S decreased with an increase in JF percentage (0.75%) in the HPJF-RC. Improved post-crack energy absorption of split tensile property, total energy absorption of split tensile property, and toughness indexes of split tensile property are the outcomes of the bridging effect of fibers [41,83].

Bangi and Horiguchi reported that the addition of 6 mm PF in composite enhanced the S-T-S [84]. Another research study revealed that a basalt fiber hybrid of 6 mm and 12 mm lengths and a total of 1% fiber content improved the S-T-S up to 34% [85]. The study reported that a JF addition to concrete improved the S-T-S of the JFRC by 13% [43]. Combining a higher fiber percentage results in the formation of air voids and a decrease in workability, which may eventually result in less compaction and a decrease in S-T-S. The lower S-T-S can be attributed to the lower amount of cement that results from incorporating a greater fiber percentage into the concrete. It is also important to mention that the S-T-S minimum and maximum coefficient of variance (CoV) percentages were 2% and 8%, respectively, as demonstrated in Table 7. From the CoV, it is evident that the S-T-S exhibits a maximum error variability of up to 8%. All these absorbed energy values and toughness indexes of S-T-S were calculated by defined methods [36,47,79].

The following empirical relation is established between the S-T-S and C-S of the HFRC:

S-T-S = F × C-S(2)

where F is the conversion factor incorporating the combined effects of fiber type, length, and content.

The energy absorbed per unit volume of material, which is also referred to as the energy absorption ability, was described as the area under the load-deflection curve, and Jules (J) are the units [81]. The SPEA1 of HPJF-1 to HPJF-9 are increased by 2.1, 0.8, 3, 1.4, 0.5, 2.4, 1.9, 0.8, and 3.7 times, respectively, as compared with that of the CM. No SPEA2 occurred in the case the of CM because under peak loading, the CM broke apart into two pieces. Therefore, only HPJF-RC has an indicate SPEA2. The SPEA2 of all the HPJF-RC specimens increased 100% as compared with that of the CM. The HPJF-RC may also be predicted to withstand split tensile loading for slightly longer than CM. The addition of fibers to concrete serves as a crack arrestor and prevents cracks from propagating. The S-TIs of HPJF-1 to HPJF-9 were greater than that of the CM. The HPJF-9 has the maximum S-TI due to its maximum pre-crack and post-crack energies. This may be because of the incorporation of the hybridaztion of one longer length of JF and one shorter length of PF, which enhanced the prevention of cracks after split tensile loading compared with other shorter fiber combinations. The loading rate all through the test was similar. The reason behind the nonlinear pre-crack behavior of concrete is the addition of hybrids of varying fiber lengths. The improved properties are due to the MSHF, i.e., polypropylene fibers, jute fibers, and silica fume, which offers crack resistance at the macro-, meso-, and micro-levels, respectively.

### 3.3. Water Absorption, Linear Shrinkage, and Mass Loss of Concrete Composites

The mass of water absorbed by the specimen divided by the specimen’s actual mass after oven-drying yields liquid transportation through capillary action, which is known as water absorption (ASTM standard C642-13). Table 8 provides information on the water absorption of the CM and all the HPJF-RC specimens. It is evident that the CM has the lowest water absorption value and that HPJF-5 has the maximum water absorption value. Water-absorbing properties are retained by natural fibers such as jute fiber. Based on this, all HPJF-RC mixtures have a higher water absorption capacity than that of the CM. Additionally, it should be noted that HPJF-RC specimens absorb more water when the length of their jute fibers increases. An investigation was conducted on the dynamic TGA curves for natural fibers (i.e., jute, kenaf, and curaua). The first decomposition began at about 220 °C to 230°C, following the initial weight loss obtained by the vaporization of moisture that was absorbed by the fiber, and it was discovered that the range of 320 °C to 335 °C represented the starting temperature for the maximum decomposition, which was linked to cellulose degradation in the structure [86]. Following the maximum decomposition, there was a gradual weight decrease related to lignin degradation, which requires a higher condensation temperature [87]. Furthermore, a weight loss of approximately 80% was noted for jute, kenaf, and curaua fiber between 150 °C and 700 °C. It was determined that significant degradation of the fibers begins at about 220–230℃, and that natural fibers are known to degrade after about 200 °C [88].

Table 8 shows the linear shrinkage for the CM and HPJF-RC. Comparing HPJF-RC with CM, an overall reduction was observed in the linear shrinkage values. It is evident that the linear shrinkage decreases with increasing jute fiber length from HPJF-7 to HPJF-9. Table 8 provides the mass loss of the CM and HPJF-RC. It is evident that the temperature of 100°C is the point at which the greatest mass loss happened. HPJF-4 had the maximum mass loss, while the CM had the minimum mass loss. It is noteworthy that the utilization of longer JFs, specifically those of 12 mm in length, had a favorable impact on mass loss.

### 3.4. Scanning Electron Microscopy (SEM) Analysis of HPJF-RC

**Figure 5:**
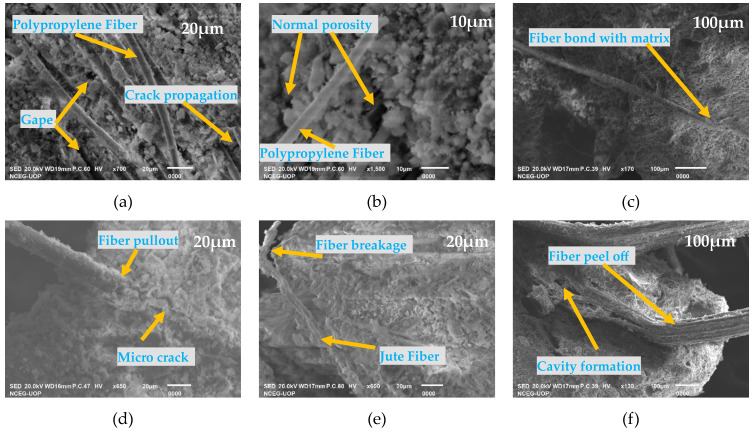
SEM analysis of the HPJF-RC fracture surface: (**a**) Proper bonding; (**b**) Embedded in concrete; (**c**) Polypropylene fiber with no breakage; (**d**) Micro-crack; (**e**) Jute fiber fractured in the concrete; and (**f**) Splitting of the fiber and pull out.

The SEM images of the tested failure surface of the HPJF-RC specimen are shown in Figure 5. These images were used to study the interfacial bonding between the hybrid fibers and the concrete matrix. Scratches on the fiber surface resulted from the removal of concrete mass beneath the embedded fibers. The initial condition of the polypropylene fibers, which had surface roughness and a diameter of 18 μm, was observed. Increased fiber content causes the mix to become more heterogeneous, which could lead to poor bond strength between the fibers and matrix and, ultimately, poor strength properties.

Figure 5a shows that there was a gap between the fiber and the concrete matrix; however, as the fiber was continuously embedded in the matrix, the gap’s width gradually minimized and eventually vanished. Due to the rough surface, some of the concrete fragments are still attached to the pulled-out polypropylene fibers in Figure 5b, where tiny pieces of concrete still connect the concrete matrix to the fiber surface. Figure 5c,d demonstrate that more hydrate stuck to the pulled-out fiber’s surface, with the raised surface showing the significant attainment of hydrate. The voids are quite small and low in the surroundings of the fibers, revealing an improved bond between the fiber and the concrete matrix. Figure 5c,e,f make it evident that the cement hydration is more adequate, the cement paste is more compact, and the polypropylene and jute fibers are dispersed randomly throughout the matrix. The distribution of jute and polypropylene fibers in the matrix, as well as the connection between size and scale, are visible among them. Better bonding between the fiber and concrete matrix is indicated by the shallow cavity surrounding the fiber. As a result of the application of load, we can also state that there is a strong bond between the fiber and the concrete matrix. Therefore, it can be said that fiber pull-out is the primary cause of fiber damage under different loads.

## 4. Optimization of the HPJF-RC

The maximum and minimum values obtained from both the mechanical and dynamic tests are shown in Table 9. It is noted that for the compressive members, such as columns, HPJF-9 with polypropylene fiber of 6 mm length and 12 mm jute fiber length is recommended because it is noted that high compressive strength is only in one of the HPJF-RC mixes, and all the other combinations have less strength as compared with the CM. For the members in which the flexural and tensile forces are dominant, HPJF-9 is recommended due to its highest flexural and split tensile strengths. The addition of hybrid artificial and natural fibers positively influences the split tensile and flexural strength. Besides this, hybrid natural fibers negatively impact the compressive strength and flexural and split tensile properties of concrete when a higher percentage and longer length of fiber are added because it creates fiber congestion, which leads to a decrement in the properties of the HPFF-RC. It is also illustrated in Table 9 that the of all the HPJF-RC mixes, the HPJF-9 hybrid composition is recommended for durability and canal lining applications.

## 5. Practical Implementation

Concrete materials are typically accessible within the proximity of the local farmers, and concrete is generally used for canal lining to minimize seepage loss [13]. The enhanced performance of concrete canal linings may assist in reducing irrigation water loss [17]. Variations in temperature, external loads, uneven foundation settlements, and thermal stress are the reasons behind those cracks. Researchers have investigated what might lead to cracks in the concrete canal lining. Crack-causing factors were categorized in accordance with the information collected. According to the findings, external loads, temperature variations, uneven foundation settling, expansion deformation of the foundation soil, humidity, and other factors were all associated with concrete cracking [89].

### 5.1. Linking HPJF-RC Properties with Canal Lining Design and Performance

Many parameters, including tensile strength, differential settlement, permeability, shrinkage, and water absorption, may influence the cracking rate in concrete canal linings [89]. The shrinkage-related tensile stresses are less than the concrete’s tensile strength, so the shrinkage-related cracking can be avoided. This indicates that its tensile strength is a critical factor in preventing shrinkage cracks in concrete. Concrete canal linings deteriorate more quickly with a rise in the water absorption rate [90]. A concrete structure experiences bending stresses as a result of differential settlement. Differential settlement-related cracking can be prevented if the concrete’s flexure strength, also known as its bending strength, is higher than the bending stresses. It is necessary to consider the role that concrete’s flexure strength plays in restraining the rate at which bending cracks occur. One factor that accelerates the cracking rate is the brittle nature of concrete. Therefore, it is necessary to improve concrete’s toughness and post-crack energy absorption to achieve a ductile and tough failure mode. In order to slow down the rate at which canal linings crack, it is crucial to investigate materials with lower shrinkage and water absorption as well as improved mechanical properties, particularly tensile and flexure strengths, and toughness.

The experimental behaviors of the CM and HPJF-RC for controlling the cracking rate in canal linings were investigated in this study. Compared with other investigated concrete mixes, HPJF-9 exhibited higher C-S, F-S, and S-T-S values. When comparing HPJF-9 with other CM and HPJF-RC mixes, an increase was observed in C-TI, F-TI, and S-TE, values. Reduced values of W-A and L-S were witnessed in the case of HPJF-9 as compared with other explored hybrid concrete mixes. Therefore, in the case of HPJF-9, the rate of cracking in the canal lining may be lower; in summary, this is likely to enhance the canal lining’s performance in terms of reduction in water losses. It is also important to mention that the HPJF-9 also showed improved values of C-TI, F-TI, CTEA, FTEA, and STEA, and HPJF-4 had improved S-TI values compared with the CM. This demonstrates that because of their superior post-crack behavior and high post-crack energy absorption capabilities, HPJF-RC can be a better option in comparison with the CM.

### 5.2. Controlling Defects in Canal Linings

In most cases, the crack in the concrete canal lining affects the entire depth of the section. The various factors that cause cracking also affect the direction and length of the cracks. In comparison with cracks resulting from other causes, the length of the cracks caused by bending and shear stresses is greater. Usually, deep cracks have a wider width. The higher rate of deterioration caused by increased water absorption might be the cause of these cracks. The compressive, tensile, and flexural strengths of concrete are the characteristics that can improve the performance of the concrete canal lining. Among these, concrete’s tensile strength is essential for preventing cracks. By handling the rate of cracking in the canal lining, its improved performance can be assured. Minimizing and controlling crack formation is a key strategy in decreasing water loss in the canal lining. Over time, the micro-cracks that initially occur in the canal lining become macro-cracks, allowing water to seep through the lining and accelerating the process of water loss. One crucial strategy for lowering the rate at which the canal lining cracks is to increase the tensile strength of the concrete [91].

Adding fibers to composite materials, such as cement paste, mortar, or concrete, can improve various mechanical properties, including flexure strength, tensile strength, fatigue-resistant strength, abrasion, and thermal impact [43,45]. Concrete that has fibers incorporated into it functions as a crack arrester [79]. Several researchers examined and claimed that jute fibers carried a large amount of the tensile stress in the composite material, acted as crack arresters, and absorbed a significant amount of energy after cracks occurred. Researchers have looked into the idea that jute fibers serve as crack arresters, absorbing a considerable amount of energy after the crack’s occurrence, and carrying a significant portion of the tensile stress in the composite material. Research was carried out on the application of concrete reinforced with polypropylene fibers for canal linings. The influence of PF on the shrinkage and crack resistance of concrete was examined for this purpose. A comparison was made between the properties of PC and concrete reinforced with polypropylene fibers. A study revealed that adding PF to concrete improved its toughness, frost resistance, impermeability, and split tensile and axial tensile strengths. The addition of polypropylene fibers to concrete has proven to be an effective strategy for preventing and reducing the occurrence of cracks in the material. This enhancement significantly improves the mechanical properties of concrete, making it a promising solution for canal lining applications. Canal lining cracking can be minimized by controlling linear shrinkage and strengthening the mechanical characteristics of the concrete [42].

## 6. Conclusions

In this study, a different type of fiber hybridization i.e., with one artificial fiber and one natural fiber, was considered to control the cracking in concrete canal linings. Water loss occurs in earth channels, mainly due to erosion and the permeability of the material. Instead of other properties, the most crucial element in crack control is the split tensile strength of concrete. The detailed behavior of HPJF-RC for the application of canal lining, including its compressive, flexural, and split tensile properties, water absorption, linear shrinkage, mass loss, etc., has not yet been studied. In this experimental work, a total of nine mixes were made with different lengths and contents of HPJF-RC, as well as a control mix. An SEM analysis was carried out to explore the hybrid fiber cracking mechanism and the bonding of the fibers with the concrete. Reducing water loss in canal linings using contemporary materials is the overarching objective of the current research. The control mix and the combination of two fibers in concrete and with different fiber contents and fiber length were studied to find an optimized hybrid fiber-reinforced concrete. In addition to this, stress–strain curves and load-deflection curves under flexure loading and split tensile loading were determined. Moreover, the strength, energy absorption, and toughness indexes under compressive, flexural, and spilt tensile loading were calculated. HPJF-RC water absorption, linear shrinkage, slump, density, and dynamic properties were also reported. The concluding remarks are as follow:

- The mix design ratio of 1:2:3 with a w/c ratio of 0.40 was used for all concrete mixes of HPJF-RC and the CM. The inclusion of hybrids of PF and JF resulted in low slump values.
- Dynamic properties of the CM and HPJF-RC showed improvement with the hybridization of PF and JF incorporated into different mixes.
- It can be noted that HPJF-9 showed the maximum C-S value in comparison with all types of HPJF-RC and the CM. When the PF content was increased up to 0.75% with a length of 6 mm and JF 0.25% (total of 1% by concrete mass) with a 12 mm length in HPJF-RC, the MSHF crack-arresting mechanism and the SF effect on the C-S increased up to 15% as compared with that of the CM. There was a 14% increase in the value of CPEA1 for HPJF-9 with a PF content 0.75% and a 6 mm length. The CPEA2 of HPJF-1 to HPJF-9 was increased by 1.08, 3.42, 1.23, 4.20, 1.80, 4.42, 0.84, 2.09, and 1.96 times as compared with the CM. The C-TI values for all the HPJF-RC specimens were greater than that of the CM.
- The F-S values of HPJF-1, HPJF-6, HPJF-7, and HPJF-9 increased by 17%, 3%, 6%, and 62% as compared with that of the CM. The HPJF-9 had the maximum F-S value. This may be due to the presence of a positive synergy effect of the hybrid PF and JF. The improvement in F-S for all the HPJF-RC specimens is due to the fiber effect of bridging.
- When the PF content was increased up to 0.75% with a length of 6 mm and the JF 0.25% with a 12 mm length in the HPJF-RC, the MSHF crack-arresting mechanism and the SF effect on the F-S increased up to 62% as compared with CM. F-S decreased with an increase in the JF percentage up to 0.75% in HPJF-RC. The FPEA1 of HPJF-1 to HPJF-4, and HPJF-6 to HPJF-9 were increased by 0.7, 0.01, 1.1, 0.3, 0.8, 0.8, 0.03, and 1.5 times, respectively, as compared with that of CM. No FPEA2 occurred in the case of the CM because under the peak loading, the CM broke apart into two pieces. Therefore, only HPJF-RC has indicated an FPEA2. The HPJF-RC may also be predicted to withstand flexural loading for slightly longer than CM. The F-TI values of HPJF-1 to HPJF-9 were greater than that of CM. The HPJF-9 had the maximum F-TI value due to its maximum pre-crack and post-crack energies.
- The S-T-S was increased by 111%, 61%, 87%, 39%, 5%, 8%, 126%, 92%, and 147% as compared with that for the CM. The HPJF-9 had the maximum value of the S-T-S. This may be due to optimum length combinations of the polypropylene and jute fibers. The overall increase in S-T-S of all the HPJF-RC specimens is due to the bridging effect of fiber hybridization. Shorter fibers, like the 6 mm length, arrest small cracks, and longer fibers, like the 12 mm length, arrest larger cracks. The SPEA1 of HPJF-1 to HPJF-9 were increased by 2.1, 0.8, 3, 1.4, 0.5, 2.4, 1.9, 0.8, and 3.7 times, respectively, as compared with that of the CM. No SPEA2 occurred in case of the CM because under peak loading, the CM broke apart into two pieces. The SPEA2 of all HPJF-RC increased 100% as compared with that of CM. The addition of fibers to concrete serves as a crack arrestor and prevents cracks from propagating. The S-TI values of HPJF-1 to HPJF-9 were greater than that of the CM. The improved properties are due to the MSHFs, i.e., PF, JF, and SF, which offered crack resistance at the macro-, meso-, and micro-level, respectively.
- After detailed experimental study, it was found that the optimized HPJF-RC is HPJF-9, with a content of 1% with a 6 mm PF and 12 mm JF proportion mix.
- The SEM images have demonstrated the positive contribution related to the strength point of view and show a good enough bond between the hybrid fibers and the concrete matrix.

Further study should be carried out on the behavior of HPJF-RC with the exploration of PF and JF content and different lengths for canal lining applications.

## Figures and Tables

**Table 1:** Details of polypropylene fiber properties provided by the manufacturer and jute fiber properties [45].

Fiber Type	Density (Kg/m^3^)	Length (mm)	Diameter (µm)	Water Absorption	Melt Point	Tensile Strength (MPa)	Alkali Resistance (%)
Polypropylene	910	6 and 12	18	Nil	160 °C	300–450	100
Jute	1300–1490	6 and 12	20–200	−	−	320–800	−

**Table 2:** Chemical composition of cement and stilica fume obtained from the manufacturers.

Composition %	SiO_2_	Al_2_O_3_	Fe_2_O_3_	CaO	MgO	SO_3_	Na_2_O	K_2_O	LOI
Cement	23.4	5.7	4.1	64.45	2.2	2.8	0.4	0.5	0.61
Silica fume	90–94	1.12	0.07	0.67	0.01	−	−	−	1.37

**Table 3:** Representation of the mix proportions of all concrete mixes of the CM and HPJF-RC.

Concrete Type	Ingredients (kg/m^3^)				Fiber Combination (wt. %)		Fiber Length (mm)		SP (%)	SF (%)	Slump (mm)	Hard Density (kg/m^3^)
	Cement	Sand	Aggregate	Water	PF	JF	PF	JF				
CM	390	780	1170	137	-	-	-	-	1.5	10	105	2312
HPJF-1	390	780	1170	137	0.50	0.50	6	6	1.5	10	81	2155
HPJF-2	390	780	1170	137	0.25	0.75	6	6	1.5	10	55	2207
HPJF-3	390	780	1170	137	0.75	0.25	6	6	1.5	10	94	2124
HPJF-4	390	780	1170	137	0.50	0.50	12	12	1.5	10	30	2133
HPJF-5	390	780	1170	137	0.25	0.75	12	12	1.5	10	25	2174
HPJF-6	390	780	1170	137	0.75	0.25	12	12	1.5	10	40	2087
HPJF-7	390	780	1170	137	0.50	0.50	6	12	1.5	10	75	2142
HPJF-8	390	780	1170	137	0.25	0.75	6	12	1.5	10	40	2188
HPJF-9	390	780	1170	137	0.75	0.25	6	12	1.5	10	82	2111

Note: 1—CM denotes the control mix, and HPJF represents the hybrid polypropylene jute fiber-reinforced concrete. 2—PF represents the polypropylene fiber; JF represents the jute fiber. 3—SF represents the silica fume as 10% of the cement, and SP represents the superplasticizer as 1.5% of the cement.

**Table 4:** Dynamic properties of the CM and HPJF-RC cylinder specimens and beamlet specimens.

Concrete Specimens		No. of Specimens for Average	Studied Parameters			
			Resonance Frequency			Dynamic Elastic Modulus Ed, (GPa)
			LRF (Hz)	TRF (Hz)	RRF (Hz)	
Cylinders	CM	6	7422 ± 140	6065 ± 233	6344 ± 250	20 ± 0.9
	HPJF-1	6	6147 ± 107	4726 ± 99	4945 ± 72	13 ± 0.5
	HPJF-2	6	4853 ± 84	3870 ± 81	4113 ± 60	8 ± 0.4
	HPJF-3	6	6337 ± 110	4923 ± 103	5151 ± 75	15 ± 0.6
	HPJF-4	6	5392 ± 94	4082 ± 86	4311 ± 63	10 ± 0.7
	HPJF-5	6	3821 ± 66	3416 ± 72	3611 ± 53	5 ± 0.3
	HPJF-6	6	5664 ± 98	4306 ± 90	4491 ± 66	10 ± 0.3
	HPJF-7	6	5840 ± 101	4490 ± 94	4698 ± 69	12 ± 0.5
	HPJF-8	6	4222 ± 73	3614 ± 76	3883 ± 57	6 ± 0.3
	HPJF-9	6	6601 ± 115	5129 ± 108	5366 ± 78	16 ± 0.5
Beamlets	CM	3	3112 ± 91	2399 ± 86	2529 ± 57	18 ± 1.1
	HPJF-1	3	2694 ± 13	2173 ± 91	2374 ± 104	12 ± 0.6
	HPJF-2	3	2076 ± 10	1640 ± 78	1715 ± 61	7 ± 0.2
	HPJF-3	3	2786 ± 13	2279 ± 82	2364 ± 103	13 ± 0.2
	HPJF-4	3	2332 ± 11	1885 ± 90	1927 ± 69	10 ± 0.1
	HPJF-5	3	1756 ± 8	1388 ± 66	1420 ± 51	5 ± 0.1
	HPJF-6	3	2380 ± 11	1885 ± 90	2095 ± 75	10 ± 0.2
	HPJF-7	3	2559 ± 12	2072 ± 99	2277 ± 82	11 ± 0.3
	HPJF-8	3	1951 ± 9	1526 ± 73	1561 ± 56	6 ± 0.2
	HPJF-9	3	2872 ± 14	2399 ± 86	2463 ± 108	15 ± 0.2

**Table 5:** CS, CPEA1, CPEA2, CTEA, and C-TI values of all the batches of concrete mixes.

Parameters	Concrete Type									
	CM	HPJF-1	HPJF-2	HPJF-3	HPJF-4	HPJF-5	HPJF-6	HPJF-7	HPJF-8	HPJF-9
C-S (MPa)	28.20 ± 0.58 (2)	27.19 ± 0.908 (3)	21.57 ± 0.7928 (4)	27.99 ± 0.530 (2)	23.44 ± 0.561 (2)	17.39 ± 0.5060 (3)	23.47 ± 0.261 (1)	25.19 ± 1.450 (6)	18.18 ± 0.634 (3)	32.51 ± 0.29 (1)
CPEA_1_ (MJ/m^3^)	0.17 ± 0.0063 (4)	0.16 ± 0.003 (2)	0.16 ± 0.0048 (3)	0.15 ± 0.006 (4)	0.14 ± 0.005 (3)	0.01 ± 0.0055 (5)	0.20 ± 0.013 (11)	0.15 ± 0.007 (4)	0.12 ± 0.002 (2)	0.20 ± 0.01 (4)
CPEA_2_ (MJ/m^3^)	0.01 ± 0.0003 (5)	0.08 ± 0.004 (5)	0.03 ± 0.0009 (3)	0.09 ± 0.007 (7)	0.04 ± 0.001 (4)	0.02 ± 0.0004 (2)	0.04 ± 0.008 (21)	0.07 ± 0.006 (9)	0.02 ± 0.0005 (2)	0.14 ± 0.01 (6)
CTEA (MJ/m^3^)	0.18 ± 0.0061 (3)	0.24 ± 0.007 (3)	0.19 ± 0.0057 (3)	0.24 ± 0.005 (2)	0.18 ± 0.006 (3)	0.14 ± 0.0051 (3)	0.16 ± 0.011 (7)	0.22 ± 0.012 (6)	0.14 ± 0.002 (1)	0.34 ± 0.002 (1)
C-TI	1.04 ± 0.0032 (0.31)	1.51 ± 0.019 (1.3)	1.20 ± 0.0004 (0.04)	1.62 ± 0.063 (4)	1.25 ± 0.004 (0.31)	1.16 ± 0.0122 (1)	1.32 ± 0.084 (6)	1.43 ± 0.021 (1)	1.18 ± 0.006 (0.5)	1.74 ± 0.07 (4)

Note: The values in () show the CoV in percentages.

**Table 6:** F-S, FPEA1, FPEA2, FTEA, and F-TI values of all batches of the concrete mixes.

Parameters	Concrete Type									
	CM	HPJF-1	HPJF-2	HPJF-3	HPJF-4	HPJF-5	HPJF-6	HPJF-7	HPJF-8	HPJF-9
F-S (MPa)	5.84 ± 0.21 (4)	6.85 ± 0.52 (8)	4.95 ± 0.19 (4)	7.95 ± 0.31 (4)	5.23 ± 0.36 (7)	3.63 ± 0.23 (6)	6.02 ± 0.42 (7)	6.17 ± 0.47 (8)	4.01 ± 0.21 (5)	9.47 ± 0.42 (4)
FPEA1 (J)	3.05 ± 0.21 (7)	5.04 ± 0.27 (5)	3.07 ± 0.23 (7)	6.45 ± 0.90 (14)	3.97 ± 0.32 (8)	0.12 ± 0.12 (4)	5.35 ± 0.52 (10)	5.53 ± 0.33 (6)	3.13 ± 0.25 (8)	7.52 ± 0.63 (8)
FPEA2 (J)	0 ± 0 (0)	7.43 ± 0.38 (5)	3.00 ± 0.24 (8)	6.45 ± 0.64 (10)	4.43 ± 0.50 (11)	1.81 ± 0.11 (6)	6.30 ± 0.32 (5)	7.01 ± 1.47 (21)	2.63 ± 0.28 (11)	14.99 ± 0.31 (2)
FTEA (J)	3.05 ± 0.21 (7)	12.47 ± 0.64 (5)	6.07 ± 0.47 (8)	16.34 ± 1.52 (9)	8.41 ± 0.82 (10)	4.55 ± 0.22 (5)	11.64 ± 0.84 (7)	12.54 ± 1.74 (14)	5.76 ± 0.39 (7)	22.51 ± 0.94 (4)
F-TI	1.00 ± 0.00 (0)	2.47 ± 0.03 (1)	1.98 ± 0.01 (0.40)	2.54 ± 0.11 (4)	2.11 ± 0.04 (2)	1.66 ± 0.01 (1)	2.18 ± 0.06 (3)	2.26 ± 0.22 (10)	1.84 ± 0.11 (6)	3.00 ± 0.13 (4)

Note: The values in () show the CoV in percentages.

**Table 7:** S-T-S, SPEA_1_, SPEA_2_, STEA and S-TI values of all batches of the concrete mixes.

Parameters	Concrete Type									
	CM	HPJF-1	HPJF-2	HPJF-3	HPJF-4	HPJF-5	HPJF-6	HPJF-7	HPJF-8	HPJF-9
S-T-S (MPa)	1.615 ± 0.03 (2)	3.42 ± 0.16 (5)	2.25 ± 0.14 (6)	4.06 ± 0.19 (5)	2.60 ± 0.21 (8)	1.70 ± 0.08 (4)	3.10 ± 0.20 (6)	3.01 ± 0.24 (8)	1.75 ± 0.10 (6)	4.79 ± 0.22 (5)
SPEA_1_ (J)	0.801 ± 0.14 (17)	2.44 ± 0.13 (5)	1.43 ± 0.13 (9)	3.17 ± 0.23 (7)	1.96 ± 0.14 (7)	0.14 ± 0.14 (11)	2.75 ± 0.25 (9)	2.33 ± 0.24 (10)	1.40 ± 0.11 (8)	3.80 ± 0.33 (9)
SPEA_2_ (J)	0 ± 0 (0)	0.73 ± 0.13 18)	0.56 ± 0.06 (11)	3.17 ± 0.07 (2)	2.18 ± 0.22 (10)	1.31 ± 0.10 (7)	2.60 ± 0.17 (7)	1.87 ± 0.16 (9)	0.99 ± 0.09 (9)	3.38 ± 0.28 (8)
STEA (J)	0.96 ± 0.14 (14)	3.18 ± 0.24 (8)	1.99 ± 0.19 (10)	4.35 ± 0.30 (7)	4.14 ± 0.36 (9)	2.50 ± 0.16 (6)	5.35 ± 0.42 (8)	4.20 ± 0.40 (10)	2.39 ± 0.20 (8)	7.18 ± 0.61 (9)
S-TI	1 ± 0 (0)	1.30 ± 0.04 (3)	1.39 ± 0.01 (0.7)	1.37 ± 0.01 (1)	2.11 ± 0.03 (1)	2.10 ± 0.15 (7)	1.95 ± 0.03 (2)	1.81 ± 0.01 (1)	1.70 ± 0.01 (1)	1.89 ± 0.01 (1)

Note: The values in () show the CoV in percentages.

**Table 8:** Water absorption, mass loss, and linear shrinkage of the CM and HPJF-RC are presented in the following table.

Specimens	Water Absorption (%)	Linear Shrinkage (%)	Mass Loss at 100 °C (%)
CM	2.08	1.49	0.5
HPJF-1	4.42	0.82	3.1
HPJF-2	5.99	0.91	3.9
HPJF-3	2.48	0.77	2.6
HPJF-4	5.56	1.14	5.0
HPJF-5	7.13	1.23	4.7
HPJF-6	3.58	1.00	4.4
HPJF-7	4.84	0.51	2.2
HPJF-8	6.38	0.66	2.6
HPJF-9	2.25	0.36	1.7

**Table 9:** Optimum mixes and recommended HPJF-RC with regard to CS, F-S, and S-T-S of all the batches.

Concrete Type	Compressive			Flexural			Split Tensile			Dynamic	Water Absorption	Linear Shrinkage
	C-S (MPa)	CTEA(J)	C-TI (−)	F-S (MPa)	FTEA (J)	F-TI (−)	S-T-S (MPa)	STEA (J)	S-TI (−)	Ed (MPa)	W-A (%)	L-S (%)
CMs values	28.2 ± 0.58 (2)	0.18 ± 0.01 (3)	1.04 ± 0.00 (0.3)	5.84 ± 0.21 (4)	3.05 ± 0.21 (7)	1.00 0.0 (0)	1.61 ± 0.03 (2)	0.96 ± 0.14 (14)	1.00 ± 0.0 (0)	20 ± 0.9	2.08	1.49
HPJF-RC with maximum value	32.5 ± 0.29 (1)	0.34 ± 0.00 (1)	1.74 ± 0.07 (4)	9.47 ± 0.42 (4)	22.5 ± 0.94 (4)	3.00 ± 0.13 (4)	4.79 ± 0.22 (5)	7.18 ± 0.61 (9)	2.11 ± 0.03 (1)	16 ± 0.5	7.13	1.23
	HPJF-9			HPJF-9			HPJF-9		HPJF-4	HPJF-9	HPJF-5	
HPJF-RC with minimum value	17.4 ± 0.51 (3)	0.14 ± 0.00 (3)	1.16 ± 0.01 (1)	3.63 ± 0.23 (6)	4.55 ± 0.22 (5)	1.66 ± 0.01 (1)	1.75 ± 0.10 (6)	1.99 ± 0.19 (10)	1.30 ± 0.04 (3)	5 ± 0.3	2.25	0.36
	HPJF-5			HPJF-5			HPJF-8	HPJF-2	HPJF-1	HPJF-5	HPJF-9	
**Recommended**												
1. For specific property												
a. From strength point of view	HPJF-9			HPJF-9			HPJF-9			HPJF-9	HPJF-9	HPJF-9
	32.5 ± 0.29 (1)	0.34 ± 0.00 (1)	1.74 ± 0.07 (4)	9.47 ± 0.42 (4)	22.5 ± 0.94 (4)	3.00 ± 0.13 (4)	4.79 ± 0.22 (5)	7.18 ± 0.61 (9)	1.89 ± 0.01 (1)	16 ± 0.5	2.25	0.36
b. From toughness point of view	HPJF-9			HPJF-9			HPJF-4			HPJF-9		HPJF-9
	32.5 ± 0.29 (1)	0.34 ± 0.00 (1)	1.74 ± 0.07 (4)	9.47 ± 0.42 (4)	22.5 ± 0.94 (4)	3.00 ± 0.13 (4)	2.60 ± 0.21 (8)	4.14 ± 0.36 (9)	2.11 ± 0.03 (1)	(−)	2.25	0.36
c. From durability point of view	HPJF-9			HPJF-9			HPJF-4			HPJF-9		HPJF-9
	32.5 ± 0.29 (1)	0.34 ± 0.00 (1)	1.74 ± 0.07 (4)	9.47 ± 0.42 (4)	22.5 ± 0.94 (4)	3.00 ± 0.13 (4)	4.79 ± 0.22 (5)	7.18 ± 0.61 (9)	1.89 ± 0.01 (1)	(−)	2.25	0.36
2. For specific application												
a. For canal lining application HPJF-9	32.5 ± 0.29 (1)	0.34 ± 0.00 (1)	1.74 ± 0.07 (4)	9.47 ± 0.42 (4)	22.5 ± 0.94 (4)	3.00 ± 0.13 (4)	4.79 ± 0.22 (5)	7.18 ± 0.61 (9)	1.89 ± 0.01 (1)	(−)	2.25	0.36

Note: The values in () show the CoV in percentages.

## Data Availability

Data are presented in the article.
